# Female Mice Are Protected from Metabolic Decline Associated with Lack of Skeletal Muscle HuR

**DOI:** 10.3390/biology10060543

**Published:** 2021-06-17

**Authors:** Allison C. Stone, Robert C. Noland, Randall L. Mynatt, Samuel E. Velasquez, David S. Bayless, Eric Ravussin, Jaycob D. Warfel

**Affiliations:** Pennington Biomedical Research Center, Louisiana State University, Baton Rouge, LA 70808, USA; Allison.Stone@pbrc.edu (A.C.S.); Robert.Noland@pbrc.edu (R.C.N.); Randall.mynatt@pbrc.edu (R.L.M.); Samuel.velasquez@pbrc.edu (S.E.V.); David.bayless@pbrc.edu (D.S.B.); Eric.Ravussin@pbrc.edu (E.R.)

**Keywords:** metabolic flexibility, sexual dimorphism, HuR, skeletal muscle, insulin resistance, lipid oxidation

## Abstract

**Simple Summary:**

Metabolic flexibility describes the ability to adapt to utilization of metabolic fuels such as carbohydrates, lipids, and proteins as they become available. The RNA binding protein HuR controls this flexibility in mouse and human skeletal muscle, but the molecular mechanisms governing this process remain poorly characterized. Additionally, studies from mice indicate that HuR control of metabolic flexibility may be more essential for males than females. This is because males lacking HuR in skeletal muscle develop hallmarks of insulin sensitivity, while females have not been shown to do so. Here we examine this sexual dimorphism in mice lacking HuR in skeletal muscle. Our results reveal that lack of HuR in skeletal muscle drives increased adiposity regardless of sex, but that this increase in adiposity drives the development of insulin resistance in male animals only. Additionally, relative to male mice, the detrimental metabolic phenotype associated with HuR inhibition in skeletal muscle can be corrected by feeding of a diet heavily composed of either lipids or carbohydrates.

**Abstract:**

Male mice lacking HuR in skeletal muscle (HuR^m−/−^) have been shown to have decreased gastrocnemius lipid oxidation and increased adiposity and insulin resistance. The same consequences have not been documented in female HuR^m−/−^ mice. Here we examine this sexually dimorphic phenotype. HuR^m−/−^ mice have an increased fat mass to lean mass ratio (FM/LM) relative to controls where food intake is similar. Increased body weight for male mice correlates with increased blood glucose during glucose tolerance tests (GTT), suggesting increased fat mass in male HuR^m−/−^ mice as a driver of decreased glucose clearance. However, HuR^m−/−^ female mice show decreased blood glucose levels during GTT relative to controls. HuR^m−/−^ mice display decreased palmitate oxidation in skeletal muscle relative to controls. This difference is more robust for male HuR^m−/−^ mice and more exaggerated for both sexes at high dietary fat. A high-fat diet stimulates expression of Pgc1α in HuR^m−/−^ male skeletal muscle, but not in females. However, the lipid oxidation Pparα pathway remains decreased in HuR^m−/−^ male mice relative to controls regardless of diet. This pathway is only decreased in female HuR^m−/−^ mice fed high fat diet. A decreased capacity for lipid oxidation in skeletal muscle in the absence of HuR may thus be linked to decreased glucose clearance in male but not female mice.

## 1. Introduction

An impaired ability to switch between carbohydrates and lipids as an energy source has classically been referred to as metabolic inflexibility; and has been documented as a consistent finding in patients with insulin resistance, obesity, and diabetes [1,2]. Metabolic flexibility is generally measured by assessing changes in respiratory exchange ratio (ΔRER) between periods of feeding when carbohydrate oxidation contributes to high RER, and periods of fasting when lipid oxidation contributes to low RER [1,2]. The question of whether decreased metabolic flexibility is a cause or consequence of the development of insulin resistance is still under debate. 

There is a strong body of literature supporting the hypothesis that metabolic inflexibility is a consequence rather than a cause of metabolic disease [3,4]. Patients with type 2 diabetes have diminished cellular glucose clearance, and this phenomenon may naturally lead to a decreased ability of insulin-sensitive cells to shift toward glucose metabolism following meal feeding. This may give the appearance of a decreased flexibility when measured by whole-body RER [3,4]. However, as these studies emphasize shifts toward carbohydrate metabolism, the possibility of defects in cellular processes governing a shift toward lipid metabolism must also be considered. In fact, recent research has indicated that obesity is promoted in humans with a decreased capacity to flex metabolism following high-fat feeding [5].

To determine whether underlying molecular mechanisms which enhance metabolic flexibility exist, we previously identified participants classified as metabolically flexible or inflexible based upon changes between sleep and 24-h RER. Importantly, subjects in both groups represented a wide range of BMI and displayed no difference between groups in fasting cellular glucose uptake ability [6]. It is important to note that participants in this study were not different from flexible controls when the higher 24-h RER was compared, but rather that the lower sleep RER (when lipid metabolism is theoretically more abundant) was markedly different between groups.

Transcriptomics analysis of skeletal muscle biopsies from these participants indicated that the metabolically inflexible subjects had decreased muscle transcript levels of mRNA known to interact with the RNA binding protein HuR [6]. HuR is a ubiquitously expressed RNA binding protein that binds to and influences the translation of a variety of RNA. HuR has recently emerged as an important control point for cellular metabolic processes in kidney, skeletal muscle, and adipose tissue [6,7,8]. 

Characterization of skeletal muscle HuR knockdown in both mice and humans revealed decreased lipid oxidation as potentially contributing to metabolic inflexibility. In vitro knockdown of HuR in either mouse or human male skeletal muscle results in decreased palmitate oxidation and decreased expression of genes involved in lipid metabolism and oxidative phosphorylation [6]. Male skeletal muscle HuR knockout mice (HuR^m−/−^) display decreased metabolic flexibility following meals, increased adiposity, and insulin resistance relative to control animals. Female HuR^m−/−^ animals displayed no differences from control females in these parameters. Our results thus suggest that HuR may increase the ability of skeletal muscle to utilize dietary lipids and thus promote metabolic flexibility; and that defects in this pathway may contribute to the development of metabolic disease more potently in males than in females. 

In the present study, we sought to examine the effects of varying dietary fat on the sexually dimorphic and metabolic phenotype of HuR^m−/−^ mice. We characterized male and female HuR^m−/−^ and control mice fed a 10% fat diet (LFD) and 45% fat diet (HFD). Both male and female HuR^m−/−^ mice showed an increase in fat mass to lean mass ratio (FM/LM) relative to controls. As body weight increased for male mice, levels of blood glucose following glucose injection also increased. Intriguingly, however, female HuR^m−/−^ mice show enhanced glucose clearance following injection with glucose relative to controls despite higher FM/LM. Additionally, HuR^m−/−^ male mice showed a more drastically decreased lipid oxidation capacity in skeletal muscle relative to controls than female HuR^m−/−^ mice. As dietary fat content increased, HuR^m−/−^ male mice showed increases in the mitochondrial biogenesis transcription factor, Pgc1α. This is despite showing decreases relative to controls on both diets in the lipid metabolism transcription factor, Pparα. Levels of Pparα controlled transcripts in female HuR^m−/−^ mice are decreased relative to controls only on high fat diet. Removal of HuR from skeletal muscle may thus promote decreased lipid utilization in skeletal muscle in males more robustly than in females. This decreased lipid handling capacity may lead to increased adiposity, which promotes the development of insulin resistance in males but not in females.

## 2. Materials and Methods

### 2.1. Animals

The breeding of skeletal muscle-specific HuR-deficient mice (HuR^m−/−^) and littermate controls (HuR^fl/fl^) has been previously described [6]. Briefly, floxed elavl1 mice were purchased from Jackson Laboratories [9] (Stock # 021431) and bred to mice expressing Cre recombinase under the control of the Mlc1f promoter [10] (Jackson Laboratories, Stock # 024713) to delete HuR in skeletal muscle. All mice were on C57BL/6 background. Mice were bred and group-housed at room temperature (RT, 22–24 °C) under a 12:12 h light:dark cycle and allowed ad libitum access to food and water. Mice were either fed a High Fat Diet (HFD) consisting of 20% calories from protein, 35% calories from carbohydrates, and 45% calories from fat (Research diets D12451, New Brunswick, NJ, USA) or a Low Fat Diet (LFD) consisting of 20% calories from protein, 70% calories from carbohydrates, and 10% calories from fat (Research diets D12450H or K, New Brunswick, NJ, USA). At 20–24 weeks of age, mice were euthanized by cervical dislocation and tissues were collected, snap-frozen in liquid nitrogen, and stored at −80 °C until subsequent analyses could be performed. Mixed gastrocnemius skeletal muscle was powdered and used for all assays presented. Mice were sacrificed according to approved procedures of the Panel on Euthanasia of the American Veterinary Medical Association. 

### 2.2. Animal Procedures

Body composition was measured using a Bruker NMR Minispec (Bruker Corporation, Billerica, MA, USA). Serum and plasma collections were performed by submandibular bleeding. Behavioral and indirect calorimetry studies were done in a 16-chamber Promethion system (Sable Systems International, Las Vegas, NV, USA) on mice at 20–22 weeks of age. For these experiments, mice were singly-housed at RT (22–24 °C) under a 12:12 h light: dark cycle and allowed ad libitum access to food and water. Glucose tolerance tests were performed after a 4 h fast as previously described [11]. Briefly, after measuring baseline blood glucose levels via the tail vein, mice received a 0.2 mL intraperitoneal injection of 20% D-glucose (40 mg glucose per mouse) and blood glucose levels were subsequently monitored at 20 min, 40 min, and 60 min post-injection. Insulin tolerance tests were performed in the fed state as previously described [11]. Briefly, after measuring baseline blood glucose levels via tail vein mice received 0.04 U of insulin per mouse in 0.2 mL phosphate buffered Saline (Sigma). Blood glucose levels were subsequently monitored at 20 min, 40 min, and 60 min post-injection. Body temperatures were taken using a digital rectal thermometer.

### 2.3. RNA and DNA Isolation

RNA were extracted from 20–30 mg of powdered mouse tissue using Trizol (ThermoFisher Scientific, Waltham, MA, USA) as previously described [12]. Briefly, samples were homogenized in 300 μL Trizol, allowed to sit at RT for 5 min, and then 0.2 mL of chloroform was added. The samples were shaken vigorously for 15 s and allowed to sit at RT for 2–3 min before they were centrifuged (12,000× *g*; 15 min; 4 °C) to induce phase separation. Roughly 150 µL of the upper aqueous supernatant containing RNA was transferred to a new microcentrifuge tube whereupon 150 µL of 70% ethanol was added and the samples were vortexed. RNA was then isolated using an RNeasy kit (Qiagen, Valencia, CA, USA) with DNAse treatment per manufacturer’s instructions. RNA content and quality (260/280 ratio range 1.9–2.1) were assessed using a Nanodrop 1000. DNA was isolated using a DNA Mini Kit (Qiagen). DNA was used directly for qPCR, while RNA was used to make cDNA prior to qPCR as described below.

### 2.4. Insulin Analysis 

An ELISA kit was used for measurement of serum insulin (Crystal Chem, Elk Grove Village, IL) which was tested in both the fed and fasted state. Mice were fasted for 4 h and ~250 µL blood was drawn via submandibular bleeding. Serum was processed using BD microtainer serum collection tubes (Becton, Dickinson and Company, NJ, USA) as per the manufacturer’s recommendations. HOMA-IR was calculated as previously described [13], using the formula: [Insulin (µU/mL) × Glucose (mg/dL)]/405.

### 2.5. Quantitative RT-PCR

Total RNA from tissues was isolated as described above. cDNA was then synthesized with an iScript cDNA synthesis kit and was used for qRT-PCR with the SYBR Green system (Bio-Rad, Hercules, CA, USA). Analysis was conducted using the ΔΔCT procedure as described previously [12]. Quantification of mus musculus ppib and 18 s transcripts were used in all experiments for duel control normalization of gene expression. For mitochondrial DNA analysis, qPCR was used to determine the average expression levels of mitochondrial Cox2 and Cytb. The average expression level of these two genes was then divided by the average expression level of nuclear encoded Hbb and Gcg genes. Primer details are provided in Supplementary Table S1. 

### 2.6. Substrate Oxidation Assays

Fatty acid oxidation was measured as the liberation of ^14^CO_2_ from [1-^14^C]-palmitate. Tissue Homogenates: Mixed gastrocnemius muscle homogenates were prepared as previously described [14]. Oxidation of palmitate (200 μM) was measured over the course of 30 min in reaction media (pH 7.4) consisting of: 100 mM sucrose, 60 mM EDTA, 10 mM Tris·HCl, 10 mM K_2_HPO_4_, 80 mM KCl, 1 mM MgCl_2_·6H_2_O, 1 mM L-carnitine, 0.05 mM malate, 1 mM DTT, 0.05 mM nicotinamide-adenine dinucleotide, 2 mM ATP, and 0.05 mM CoA. Homogenates were incubated with or without 1mM pyruvate in order to measure inhibition of palmitate oxidation. 

### 2.7. Study Approval

Animal studies were conducted at Pennington Biomedical Research Center’s AALAC approved facility. All experiments were in compliance with the NIH Guide for the Care and Use of Laboratory Animals and approved by the Pennington Biomedical Research Center Institutional Animal Care and Use Committee under PBRC IACUC Protocol #1049.

### 2.8. Statistics

Data are expressed as mean ± SD. Microsoft Excel software was used for analysis of variance with paired two-tailed Student’s *t*-tests. Normality was established using GraphPad Prism software and the D’Agostino–Pearson normality test. For gene expression where sample sizes were *n* < 8 GraphPad Prism software and Mann–Whitney U Tests were performed to calculate *p*-values. JMP software from SAS was used for ANCOVA analysis. Fisher’s combined probability test was used for pathway *p*-value calculation using Microsoft Excel to calculate χ^2^ values [15]. For RER and energy expenditure (EE) measurements, two days of data were averaged together and rows were pruned by averaging every four points together for statistical smoothing. EE was calculated from VO_2_ intake and VCO_2_ output using the Weir equation (Kcal/hour = 60 × (3.94 VO_2_ + 1.11 VCO_2_) [16]. For food intake data each daily food intake amount in grams was taken as a separate data point across a six-day period and averaged together.

## 3. Results

### 3.1. Dietary Fat Content Influences Fat Mass Accumulation Relative to Controls in HuR^m−/−^ Males but Not Females

**Figure 1:**
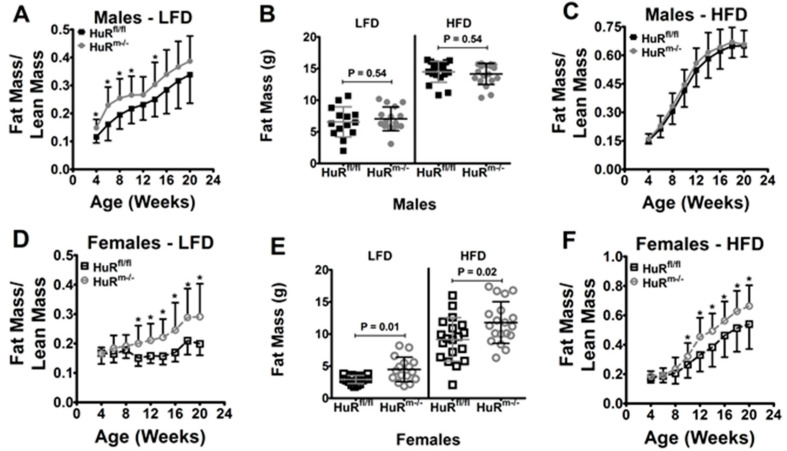
Dietary fat content affects FM/LM phenotype of HuR^m−/−^ male but not female mice. (**A**) Gain in FM/LM is shown for male control (black) (*n* = 14) or HuR^m−/−^ (grey) (*n* = 16) mice fed LFD. (**B**) Fat mass at 20 weeks of age for male control (black) or HuR^m−/−^ (grey) mice fed either LFD (*n* = 14–16) or HFD (*n* = 18–19). (**C**) Gain in FM/LM is shown for male control (black) (*n* = 18) or HuR^m−/−^ (grey) (*n* = 19) mice fed HFD. (**D**) Gain in FM/LM is shown for female control (black) (*n* = 16) or HuR^m−/−^ (grey) (*n* = 16) mice fed LFD. (**E**) Fat mass at 20 weeks of age for female control (black) or HuR^m−/−^ (grey) mice fed either LFD (*n* = 16) or HFD (*n* = 19). (**F**) Gain in FM/LM is shown for female control (black) or HuR^m−/−^ (grey) mice fed HFD (*n* = 19). * *p* < 0.05.

Our initial characterizations of male HuR^m−/−^ mice fed 25% fat diet revealed decreased glucose clearance relative to controls, potentially linked to increased obesity [6]. We thus hypothesized that as dietary fat content increased, the increased adiposity and insulin resistant phenotype in HuR^m−/−^ male mice would be further exaggerated relative to control males. HuR^m−/−^ male mice display increased fat mass and decreased lean mass relative to control animals when fed LFD with statistical significance at several timepoints throughout the study (Supplementary Figure S1A,B). Despite no difference in overall body mass (Supplementary Figure S1C), an increased fat mass to lean mass ratio (FM/LM) was observed in comparison to control males (Figure 1A). Contrary to our hypothesis, when male HuR^m−/−^ and control mice eat HFD there is no significant difference in fat mass between groups at 20 weeks of age (*n* = 18–19, *p* = 0.54) (Figure 1B). Differences in lean mass between genotypes on HFD are also less pronounced relative to LFD (Supplementary Figure S1A,B). HuR^m−/−^ male mice thus show no significant increase in (FM/LM) relative to controls throughout the 20-week study (Figure 1C).

Female HuR^m−/−^ mice show similarities to males in their increase of (FM/LM) relative to controls when fed LFD (Figure 1D). Unlike with male animals, this is due primarily to an increase in fat mass relative to control females with no decrease in lean mass noted, causing an increased overall body weight for HuR^m−/−^ females relative to controls (Supplementary Figure S1D–F). In fact, HuR^m−/−^ mice show increases in lean mass relative to controls at some timepoints on both diets. Both HuR^m−/−^ and control females show similar increases in fat mass at 20 weeks of age when fed HFD relative to LFD (Figure 1E). Unlike HuR^m−/−^ males, HuR^m−/−^ female mice thus continue to show increased (FM/LM) relative to controls when fed HFD (Figure 1F). These results indicate that removal of HuR from mouse skeletal muscle promotes greater (FM/LM) relative to controls in both males and females. However, HFD ablates this increase in male animals only.

### 3.2. Food Intake Decreases as Dietary Fat Increases in Male but Not Female HuR^m−/−^ Mice

**Figure 2:**
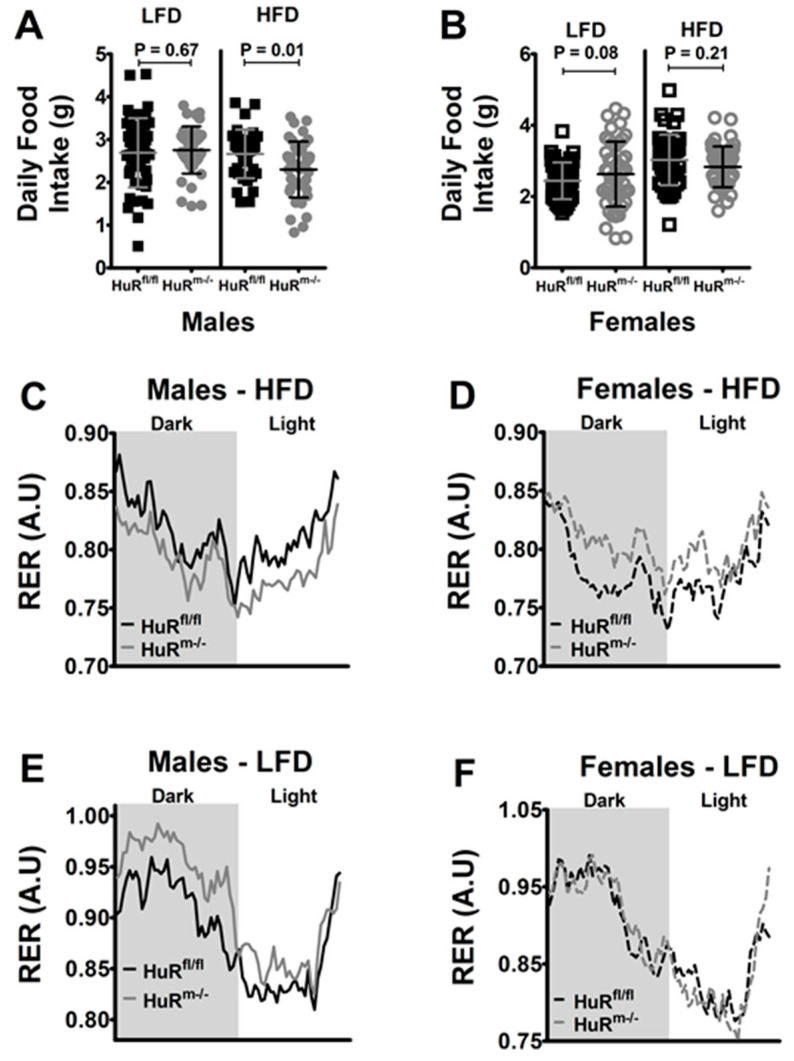
Male but not female HuR^m−/−^ mice display decreased food intake as dietary fat increases. (**A**) Average daily food intake is shown for male control (black) and HuR^m−/−^ (grey) mice fed LFD or HFD (*n* = 36–48). (**B**) Average daily food intake is shown for female control (black) and HuR^m−/−^ (grey) mice fed LFD or HFD (*n* = 48). (**C**) Average RER is shown for dark and light cycles for male control (black) and HuR^m−/−^ (grey) mice fed HFD (*n* = 12–14). (**D**) Average RER is shown for dark and light cycles for female control (black) and HuR^m−/−^ (grey) mice fed HFD (*n* = 16). (**E**) Average RER is shown for dark and light cycles for male control (black) and HuR^m−/−^ (grey) mice fed LFD (*n* = 14–16). (**F**) Average RER is shown for dark and light cycles for female control (black) and HuR^m−/−^ (grey) mice fed LFD (*n* = 16).

Our previous results suggested that HuR^m−/−^ mice maintained a higher RER than controls throughout day and night cycles despite no differences in food intake, thus indicating decreased whole-body lipid oxidation when food intake is not different [6]. We hypothesized that HuR^m−/−^ male mice may decrease food intake at higher dietary fat contents, which could lead to the suppression of fat mass differences relative to controls. HuR^m−/−^ male mice show no difference in food intake compared to controls when fed LFD, whereas they show decreased food intake relative to controls when fed HFD (Figure 2A). Female HuR^m−/−^ animals show an intriguing pattern in contrast to male animals, displaying a slight decrease relative to controls in food intake on LFD, although this decrease is only marginally significant (Figure 2B). Females fed HFD show no differences between genotypes in terms of food intake. For all animals regardless of genotype or sex, a strong correlation exists between dietary fat intake and total fat mass (Supplementary Figure S2). 

To test if these decreases in food intake were reflected in daily RER difference, we used indirect calorimetry measurements to assess changes in RER between HuR^m−/−^ and control mice. Throughout day and night cycles, male HuR^m−/−^ mice on HFD display a decreased RER relative to control animals (Figure 2C). This is a trend that does not hold true for female animals, which show increases in RER relative to controls (Figure 2D). HuR^m−/−^ male mice display RER values higher than controls at several time points when fed LFD, continuing to suggest an increased reliance on carbohydrates in agreement with previously reported results [6,17] (Figure 2E). No difference is present in RER patterns between genotypes for female animals fed LFD (Figure 2F). These results reinforce the hypothesis that the decreased food intake of HuR^m−/−^ mice relative to controls on HFD is responsible for negating the (FM/LM) differences seen on LFD.

It was previously reported that HuR^m−/−^ mice display increased energy expenditure relative to control animals, indicative of an increased oxidative phenotype in skeletal muscle [17]. HuR^m−/−^ female mice fed HFD likewise show slightly higher energy expenditure relative to control females, but only during the day period while animals are not as active. However, our results indicate no other consistent or significant differences in energy expenditure between control and HuR^m−/−^ mice regardless of sex (Supplementary Figure S3). It is notable that the reported studies of Janice-Sanchez et al. were conducted on male mice at 8–10 weeks of age fed a very low fat diet (6.2% Calories from fat). As discussed in Material and Methods in Section 2, our energy expenditure results were collected at 20–22 weeks of age, when mice are about 10 g heavier, on average. Energy expenditure is known to generally increase linearly as a function of body weight, lean mass, or fat mass [18]. It is therefore important to note that our results do not contradict these previously reported results. Instead, these studies together suggest that the metabolic consequences of HuR inhibition in skeletal muscle vary with body composition, which is dependent upon age, sex, and diet.

### 3.3. Female HuR^m−/−^ Mice Show Enhanced Glucose Clearance Relative to Controls, While Increased Fat Mass Contributes to Decreased Glucose Clearance in HuR^m−/−^ Males

**Figure 3:**
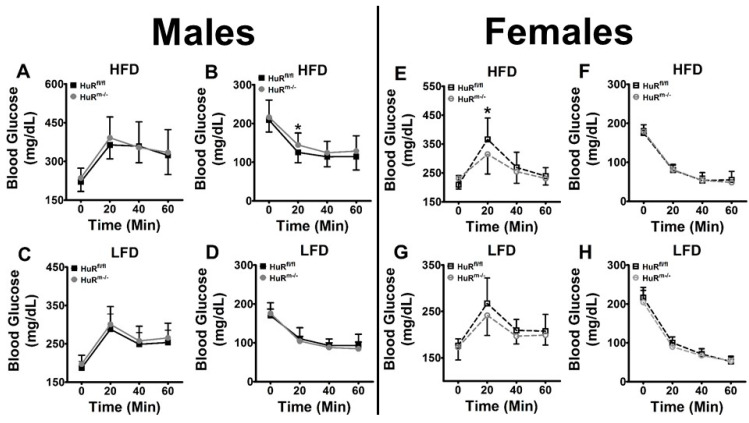
HuR^m−/−^ females but not males have increased glucose clearance relative to controls on HFD (**A**) GTT at 20 weeks of age for male control (black) or HuR^m−/−^ (grey) mice fed HFD (*n* = 19). (**B**) ITT at 20 weeks of age for male control (black) (*n* = 18) or HuR^m−/−^ (grey) (*n* = 19) mice fed HFD. (**C**) GTT at 20 weeks of age for male control (black) (*n* = 14) or HuR^m−/−^ (grey) (*n* = 16) mice fed LFD. (**D**) ITT at 20 weeks of age for male control (black) (*n* = 14) or HuR^m−/−^ (grey) (*n* = 16) mice fed LFD. (**E**) GTT at 20 weeks of age for female control (black) (*n* = 19) or HuR^m−/−^ (grey) mice fed HFD (*n* = 18). (**F**) ITT at 20 weeks of age for female control (black) (*n* = 19) or HuR^m−/−^ (grey) (*n* = 18) mice fed HFD. (**G**) GTT at 20 weeks of age for female control (black) (*n* = 16) or HuR^m−/−^ (grey) (*n* = 16) mice fed LFD. (**H**) ITT at 20 weeks of age for female control (black) (*n* = 16) or HuR^m−/−^ (grey) mice fed LFD (*n* = 16). * *p* ≤ 0.05.

We previously reported that HuR^m−/−^ males had hallmarks of increased insulin resistance relative to control animals [6]. We thus performed both glucose and insulin tolerance tests on male and female HuR^m−/−^ and control mice fed different diets to determine how changes in fat mass correlated with glucose clearance. At 20 weeks of age, male HuR^m−/−^ mice show little difference relative to controls in blood glucose levels over the course of 60 min following injection with either glucose or insulin when fed HFD (Figure 3A,B and Figure S4A,B). Male HuR^m−/−^ mice also display no statistical difference in blood glucose relative to controls during ITT and GTT when fed LFD (Figure 3C,D and Figure S4C,D). Despite the marginal increase in fat mass in HuR^m−/−^ animals relative to controls, female HuR^m−/−^ animals had decreased blood glucose during GTT on HFD and a marginally significant decrease on LFD (Figure 3E and Figure S4E). ITT results with HFD and LFD were no different between HuR^m−/−^ or control female animals (Figure 3F–H and Figure S4F–H).

**Figure 4:**
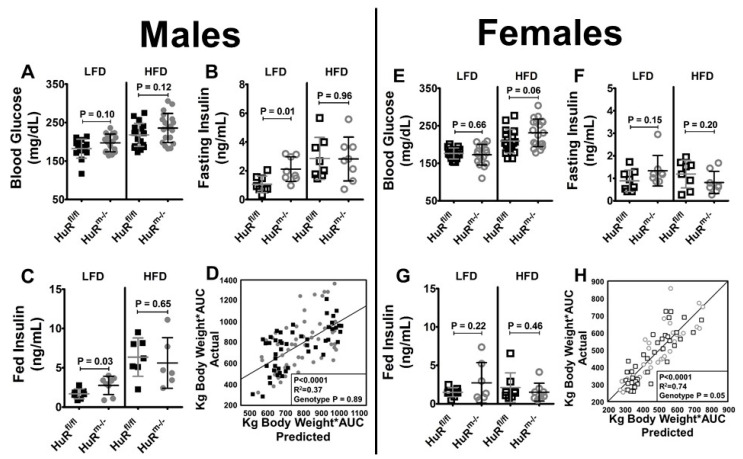
Increased fat mass drives decreased glucose clearance relative to controls in HuR^m−/−^ males only (**A**) Fasted blood glucose levels at 20 weeks of age for male control (black) or HuR^m−/−^ (grey) mice fed either LFD or HFD (*n* = 14–19). (**B**) Fasted serum insulin levels at 20 weeks of age for male control (black) or HuR^m−/−^ (grey) mice fed either LFD or HFD (*n* = 8). (**C**) Fed serum insulin levels at 20 weeks of age for male control (black) or HuR^m−/−^ (grey) mice fed either LFD or HFD (*n* = 7–8). (**D**) ANCOVA analysis showing the contribution of genotype to decrease in glucose clearance for male control (black) or HuR^m−/−^ (grey) mice as fat mass increases. (**E**) Fasted blood glucose levels at 20 weeks of age for female control (black) or HuR^m−/−^ (grey) mice fed either LFD or HFD (*n* = 16–19). (**F**) Fasted serum insulin levels at 20 weeks of age for female control (black) or HuR^m−/−^ (grey) mice fed either LFD or HFD (*n* = 8). (**G**) Fed serum insulin levels at 20 weeks of age for female control (black) or HuR^m−/−^ (grey) mice fed either LFD or HFD (*n* = 7–8). (**H**) ANCOVA analysis showing the contribution of genotype to decrease in glucose clearance for female control (black) or HuR^m−/−^ (grey) mice as fat mass increases.

We also tested if the changes in (FM/LM) between genotypes and diets were associated with increased fasting blood glucose and serum insulin levels as hallmarks of insulin resistance. HuR^m−/−^ male mice show a marginally significant increase in fasting blood glucose relative to controls regardless of diet (Figure 4A). Additionally, these mice show higher fed and fasted serum insulin levels relative to control males when fed LFD, suggesting that greater (FM/LM) may be driving the development of an insulin resistant phenotype (Figure 4B,C). Though female HuR^m−/−^ mice show no difference in fasting blood glucose relative to controls when fed LFD, these same mice show a slight increase in fasting blood glucose relative to controls when fed HFD (Figure 4E). This is despite the GTT results indicating that glucose is cleared more rapidly after injection in female HuR^m−/−^ mice (Figure 3E). Consistent with previous studies suggesting that female mice are more resistant to obesity induced insulin resistance than male animals [19,20], no differences in serum insulin levels were found between female animals regardless of genotype, diet, or fed versus fasted state (Figure 4F,G).

The results for male animals are consistent with the known relationship between increased adiposity and decreased glucose clearance [21]. We thus hypothesized that decreased glucose clearance in HuR^m−/−^ male mice relative to controls may only be observed when these animals show increased adiposity relative to controls. When GTTAUC*Kg Body Mass is plotted as a function of fat mass for all male animals, a positive correlation is displayed (Figure 4D) (R^2^ = 0.37, *p* < 0.0001). ANCOVA analysis of these data reveal that no genotype specific effect exists for this increase in fat mass promoting decreased glucose clearance (*p* = 0.89). This suggests that when fat mass is decreased in either HuR^m−/−^ or control mice, glucose clearance is increased in a similar way for both genotypes. This trend persists in female animals with an even stronger correlation (R^2^ = 0.74, *p* = 0.0001). However, ANCOVA analysis of the female data suggests that a genotype specific effect influences glucose uptake changes as fat mass increases (*p* = 0.05) (Figure 4H). These data thus suggest that female HuR^m−/−^ animals may have an enhanced capacity to clear glucose, which is not as negatively affected by increases in fat mass as in male animals.

Using fasting blood glucose and insulin, we also calculated HOMA-IR as a measure of insulin resistance. For male animals, feeding HFD significantly increases fat mass (Figure 1B) and also HOMA-IR relative to LFD regardless of genotype (Supplementary Figure S5A). Female animals do not, however, display increases in HOMA-IR as fat mass increases (Supplementary Figure S5B). Only for males fed LFD are HOMA-IR levels elevated for HuR^m−/−^ animals relative to controls. For this same cohort of animals, FM/LM is significantly elevated for HuR^m−/−^ animals at several timepoints in our study (Figure 1A). Because HOMA-IR is determined using both blood glucose and insulin levels [13], these data further support the hypothesis that HuR^m−/−^ males display increased hallmarks of insulin resistance relative to controls only when fat mass is also increased.

### 3.4. HuR^m−/−^ Mice Exhibit Decreased Lipid Oxidation Relative to Controls in Skeletal Muscle as Dietary Fat Increases More Abundantly in Males than in Females

**Figure 5:**
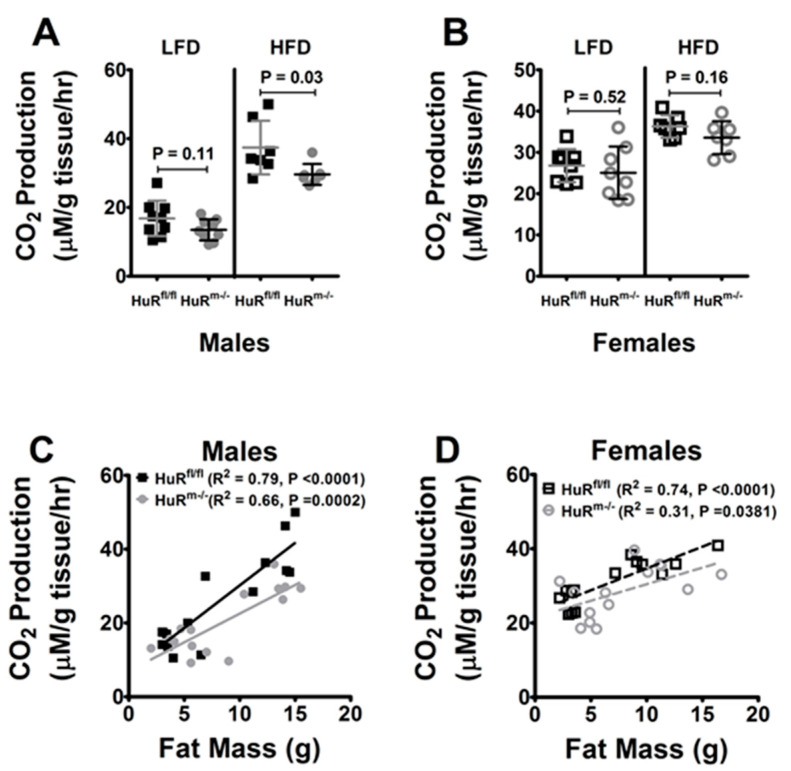
Increased dietary fat activates lipid oxidation less robustly in HuR^m−/−^ mice. (**A**) Palmitate oxidation as measured by CO_2_ production in gastrocnemius homogenates from control (black) and HuR^m−/−^ (grey) male mice fed either LFD or HFD (*n* = 7–9). (**B**) Palmitate oxidation in gastrocnemius homogenates from control (black) and HuR^m−/−^ (grey) female mice (*n* = 8–9) fed either LFD or HFD. (**C**) Palmitate oxidation in gastrocnemius as a function of fat mass for control (black) and HuR^m−/−^ (grey) male mice. (**D**) Palmitate oxidation in gastrocnemius as a function of fat mass for control (black) and HuR^m−/−^ (grey) female mice.

We have previously shown that HuR^m−/−^ male mice have lower palmitate oxidation in skeletal muscle than controls [11]. We assayed palmitate oxidation in gastrocnemius homogenates from mice fed LFD and HFD to test how diet composition altered this phenomenon. HuR^m−/−^ male mice show about a 20% decrease in palmitate oxidation relative to controls when fed HFD. Though this difference persists in mice on LFD (Figure 5A), the significance is not as robust (*p* = 0.11). Female HuR^m−/−^ mice show no difference in palmitate oxidation from controls on LFD, and only a 5% decrease relative to control females with a marginal significance of *p* = 0.16 when fed HFD (Figure 5B).

For males and females of both genotypes, lipid oxidation is increased as fat mass increases (Figure 5C,D). However, for both HuR^m−/−^ males and females, the linear increase in CO2 production as a function of fat mass is less robust. Additionally, the slope for HuR^m−/−^ females is shallower than that for HuR^m−/−^ males, suggesting a slower and more steady activation of lipid oxidation in HuR^m−/−^ females as fat mass increases. Taken together, these results may indicate that female animals have a higher capacity for activation of lipid oxidation as dietary fat increases, even in the absence of skeletal muscle HuR.

### 3.5. Skeletal Muscle Levels of Pgc1α but Not Pparα Are Increased Relative to Controls in HuR^m−/−^ Male Mice, While Female HuR^m−/−^ Mice Show Little Difference in These Markers

It has been previously reported that levels of Pparα, a transcription factor known to be an important promoter of lipid metabolism in skeletal muscle, were shown to be decreased in muscle of male HuR^m−/−^ mice, which could contribute to decreased lipid oxidation [17]. However, HuR was also shown to have the capacity to destabilize mRNA for the metabolic regulator Pgc1α in skeletal muscle [17]. We used mRNA analysis to assay for changes in the levels of Pparα and Pgc1α in skeletal muscle in order to test the hypothesis that disruptions to these transcription factors may correlate with changes to skeletal muscle lipid oxidation.

**Figure 6:**
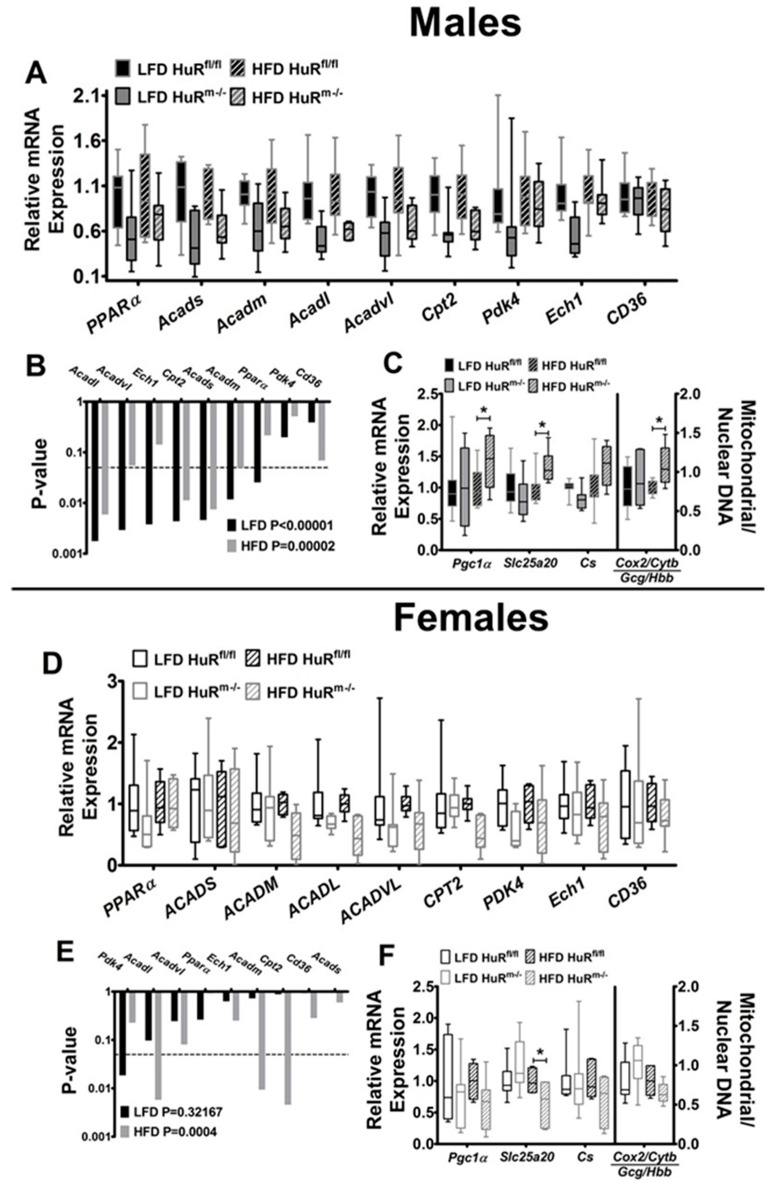
HuR^m−/−^ male but not female mice show increased mitochondrial content on HFD with decreased activation of Pparα. (**A**) Gene expression profile for transcripts controlled by the lipid oxidation transcription factor, Pparα are shown for control (black) and HuR^m−/−^ (grey) males on LFD (solid) and HFD (striped). (*n* = 7–8) (**B**) Pathway *p*-value analysis for Pparα pathway within males fed LFD (black) and HFD (grey). The dashed line indicates *p* = 0.05. (**C**) Gene expression profile for Pgc1α and its targets, including mitochondrial DNA are shown for controls (black) and HuR^m−/−^ (grey) on LFD (solid) and HFD (striped). (*n* = 5–8). (**D**) Panel A) is replicated for control (black) and HuR^m−/−^ (grey) females on LFD (open) and HFD (striped). (*n* = 6–8) (**E**) Pathway *p*-value analysis for Pparα pathway within females fed LFD (black) and HFD (grey). The dashed line indicates *p* = 0.05. (**F**) Gene expression profile for Pgc1α and its targets, including mitochondrial DNA are shown for control (black) and HuR^m−/−^ (grey) females on LFD (solid) and HFD (striped). (*n* = 6–8). * *p* ≤ 0.05.

Transcript levels of the transcription factor Pparα, which promotes transcription of lipid oxidation genes [22], are decreased in male HuR^m-/-^ skeletal muscle relative to control males regardless of diet (Figure 6A). This is in correlation with decreases to the mRNA levels of several acyl-coA dehydrogenases and other β-oxidation genes for which Pparα controls transcription (Figure 6A) [22]. Using Fisher’s combined probability test [15] our data show that regardless of diet, Pparα controlled lipid oxidation genes are collectively decreased in HuR^m−/−^ skeletal muscle relative to controls (Figure 6B). Despite this decrease in the Pparα pathway, Male HuR^m−/−^ mice show increases in Pgc1α levels in gastrocnemius muscle relative to controls when fed HFD (Figure 6C). This correlates with increases in the expression of genes for which Pgc1α promotes transcription such as Cs and Slc25a20 (Figure 5C) [23,24]. Pgc1α is also known to promote mitochondrial biogenesis [23], and male HuR^m−/−^ mice show increases in the ratio of mitochondrial genes Cox2 and CytB to the genomic genes Gcg and Hbb, indicating a higher mitochondrial DNA content relative to controls (Figure 6C).

Levels of Pparα show little difference in female HuR^m−/−^ mice relative to controls regardless of diet (Figure 6D). Contrary to what is seen in male animals, Pparα target genes show mixed results, with some significant decreases relative to controls, an effect that is exaggerated at higher dietary fat content (Figure 6D). Fisher’s combined probability test for Pparα controlled lipid oxidation genes shows no significant pathway decrease in female HuR^m−/−^ muscle relative to controls on LFD (Figure 6E). However, these mice on HFD display a significant decrease relative to controls in the Pparα pathway (Figure 6E). Female HuR^m−/−^ mice also do not show robust changes in Pgc1α relative to controls on either diet, but do have slight and marginally significant decreases in Pgc1α as well as in several of its target genes and in mitochondrial DNA markers relative to controls on HFD (Figure 6F). These results implicate a decreased ability to activate Pparα controlled lipid oxidation in HuR^m−/−^ mice of both sexes, which is more exaggerated as dietary fat increases. However, these results also suggest a stronger activation of lipid oxidation genes in female mice lacking skeletal muscle HuR relative to males, which could be promoting lipid oxidation more robustly than in male HuR^m−/−^ mice.

## 4. Discussion

Male and female clinical trial participants with no impairment in glucose clearance can display decreased shifts in RER from a fed to a fasted state [6]. These participants also have a common transcriptomic signature in skeletal muscle which differs from that of metabolically flexible subjects. Specifically, they display decreased transcript levels of mRNA known to interact with the RNA binding protein HuR. It is important to reiterate that these participants are different from flexible controls only in sleep RER, when lipid metabolism is theoretically more abundant [6]. This data suggests that metabolic inflexibility may be reflective of a decreased capacity to shift toward lipid metabolism; and our characterization of mice and human skeletal muscle deficient in HuR corroborated this finding by showing decreased lipid oxidation relative to controls.

These initial characterizations showed that while male HuR^m−/−^ mice were susceptible to decreased metabolic function, female HuR^m−/−^ mice displayed little difference relative to controls. In the present study we sought to better understand the sexually dimorphic consequences of HuR removal from skeletal muscle. We thus examined how alterations in dietary fat content affected the development of obesity and insulin resistance in HuR^m−/−^ male and female mice.

Both male and female HuR^m−/−^ mice have a significantly increased susceptibility to gain fat mass regardless of dietary fat content. This result is somewhat modified from our previous studies indicating that HuR^m−/−^ females had no significant differences in fat mass or blood glucose levels during GTT or ITT relative to controls [6]. Post-hoc power analysis determined that at least 14 animals per genotype would be required to detect the observed 12% difference in fat mass to lean mass ratio with an alpha error level of 5% and a statistical power of at least 80%. Unfortunately, this suggests that our initial study was underpowered. However, the results did show a trend toward higher fat mass to lean mass ratio at several time points and lower blood glucose for HuR^m−/−^ females relative to controls with a sample size of 8–10 animals per group, despite the absence of statistical significance. Though the present findings with a greater sample size per group modify our originally observed conclusions, no blatant opposition to these conclusions is present that cannot be explained by the lack of sufficient sample size.

The increased blood glucose and serum insulin levels observed for male HuR^m−/−^ animals relative to controls is directly related to the observed increase in adiposity in these animals. It is unclear at present why male HuR^m−/−^ mice decrease food intake during HFD feeding. However, even while consuming decreased dietary fat relative to control males on HFD, HuR^m−/−^ males still maintain adiposity at the same level as controls, which again suggests an increased susceptibility to fat mass accumulation. Compared to mice on 25% fat diet [6], fat mass in HuR^m−/−^ mice is not statistically different from controls at 20 weeks of age when mice eat LFD or HFD. We can thus limit the development of insulin resistance relative to controls in HuR^m−/−^ males by feeding a diet either high in carbohydrate (LFD) or high in fat (HFD).

Increased insulin resistance as a direct result of increased adiposity is a well-established finding for mice on a C57BL/6 background [25]. However, female mice are known to have decreased susceptibility to obesity induced insulin resistance [26]. Our results showing an increase in FM/LM of HuR^m−/−^ female mice relative to controls with no exaggerated impairment of insulin sensitivity are thus consistent with previously reported phenomena. Higher levels of serum adipokines within female mice and humans have been associated with better prognoses in females with obesity and metabolic inflexibility, providing a potential avenue of investigation as to the enhanced metabolic performance of female HuR^m−/−^ mice relative to males [27,28].

It is intriguing that HuR^m−/−^ female mice have decreased blood glucose levels during GTT relative to controls. It was previously reported that male HuR^m−/−^ mice have an increased oxidative capacity and exercise endurance relative to controls due to a greater prevalence of type 1 skeletal muscle fibers [17]. This is consistently accompanied by an increase in RER relative to controls, indicating greater reliance upon carbohydrates [6,17]. These findings could suggest that nutrients are disproportionately distributed amongst muscle tissues depending upon fiber types; and a full analysis of glucose and lipid uptake via radiolabeled substrate injection is essential to determine the fate of serum nutrients in the absence of skeletal muscle HuR.

Both control and HuR^m−/−^ mice increase lipid oxidation in skeletal muscle as adiposity increases, and yet increased fat accumulation does not stimulate lipid oxidation in skeletal muscle of HuR^m−/−^ mice to the degree present in controls. Though female control mice increase lipid oxidation at a slightly higher rate than female HuR^m−/−^ mice, as fat mass increases, female HuR^m−/−^ animals only show a slightly reduced lipid oxidation relative to control females over the range of fat masses tested. These results suggest that skeletal muscle lacking HuR may have a diminished capacity for activating lipid metabolism as fat becomes more readily available. However, female HuR^m−/−^ mice are not as drastically affected by this.

It is important to note that in order to compare our present studies to those previously conducted [6,17], we housed mice and performed experiments below thermoneutrality (28 °C) at room temperature (22–24 °C). The existence of cold stress on mice being housed at room temperature is a well-documented phenomenon [18,29]; and the redirecting of lipids toward brown adipose tissue for heat generation may be contributing to a portion of the observed phenotype for HuR^m−/−^ mice. Indeed, we see that female HuR^m−/−^ mice display both WAT and BAT hypertrophy relative to controls (Supplementary Figure S6B,D), which is known to be associated with higher activation of BAT [30,31]. This occurs despite no differences in body temperature for animals tested (Supplementary Figure S6E,F). Our data reveal lower skeletal muscle lipid oxidation and fat mass accumulation in HuR^m−/−^ mice. The exact fate of these lipids is however unclear; and future studies conducted at thermoneutrality are essential for decreasing the potential for BAT activation as a confounder of acquired data.

Pgc1α levels relative to controls change depending on diet in HuR^m−/−^ males, without significant corresponding changes in Pparα. Our results corroborate the previous observation that Pparα and the genes that it stimulates transcription of are decreased in muscle of HuR^m−/−^ mice relative to controls [17]. This decrease is maintained in gastrocnemius muscle regardless of the amount of dietary fat ingested. Within this muscle, Pgc1α is increased while the Pparα pathway is decreased in HuR^m−/−^ males relative to controls on HFD. Pgc1α is known to enhance mitochondrial biogenesis [23] and Pparα is known to stimulate β-oxidation in the mitochondria [32]. An increase in Pgc1α without an increase in Pparα could thus lead to the observed increase in mitochondrial content on HFD without an increase in lipid oxidation as robust as is seen in controls.

It was previously reported that fiber type may influence the degree to which Pgc1α is upregulated relative to controls in HuR^m−/−^ muscle [17]. Levels of Pgc1α were increased following knockout of skeletal muscle HuR in a fiber type specific manner, with more robust increases in Pgc1α occurring in the type 1 abundant, oxidative soleus muscle [17]. However, this effect was much less robust in type 2 tissues such as extensor digitorum longus. In the present study, we chose to analyze gastrocnemius muscle because it contains relatively equal type 1 and type 2 fibers [33,34], thus giving a general idea of how skeletal muscle performs in the absence of HuR regardless of fiber type. Future studies examining changes in lipid oxidation within separate type 1 and type 2 skeletal muscle fibers of HuR^m−/−^ mice are essential for better understanding of HuR controlled lipid metabolism.

HuR^m−/−^ may influence cellular metabolism in skeletal muscle through direct destabilization of Pgc1α mRNA [17]. However, the mechanism through which HuR promotes lipid oxidation via Pparα is still under investigation. Compared to male animals we observed smaller changes in the transcripts of the metabolic regulators Pgc1α and Pparα between control and HuR^m−/−^ females. However, HuR^m−/−^ females on HFD showed a marginally significant decrease in Pgc1α and its targets, including mitochondrial DNA. Gastrocnemius muscle in female mammals have higher mitochondrial content relative to males in general [35], and levels of Pgc1α and Pparα in females are known to be related to increases in estrogen signaling relative to males [36]. Specifically, females have been shown to have an increased capacity for lipid oxidation in skeletal muscle relative to males due to enhanced levels of estradiols [19]. Such estradiols are known to improve mitochondrial function via activation of ERα and Pparα [22]. However, previous work has shown no interaction between HuR and the estrogen receptor esr1 mRNA [37], suggesting a potential HuR independent avenue through which lipid oxidation could be upregulated in females relative to males. Further investigation into the role of estrogen’s stimulation of this pathway in the absence of HuR is thus warranted.

## 5. Concluding Summary

Collectively, our results show that HuR stimulates lipid oxidation in mouse skeletal muscle via interactions with the Pparα and Pgc1α pathways. Removal of HuR from skeletal muscle results in a greater decrease in lipid oxidation in males than in females. Regardless of sex, however, lack of skeletal muscle HuR promotes increased fat mass where food intake is similar. This increase in fat mass is a direct driver of decreased glucose clearance in male animals, but not females. HuR may thus be contributing to the storage and utilization of lipids in order to promote metabolic flexibility, which is more essential for adequate lipid oxidation in males than in females.

## Data Availability

The raw data used to generate these figures are readily available upon request.

## Supplementary Materials

The following are available online at https://www.mdpi.com/article/10.3390/biology10060543/s1, Table S1. Primers used in this study. Figure S1. Fat and Lean mass gain in control and HuR^m−/−^ mice. Figure S2. Increased dietary fat intake increases fat mass in control and HuR^m−/−^ mice. Figure S3. Energy Expenditure graphs for mice in this study. Figure S4. Area under the curve for GTT and ITT in this study. Figure S5. HOMA-IR calculated from 4-hour fasted glucose and insulin from animals in this study. Figure S6. Fat pad weights and body temperature for HuR^m^^−/−^ and control mice.
