# Supplementary material for: Female Mice Are Protected from Metabolic Decline Associated with Lack of Skeletal Muscle HuR
Source: Biology (Basel). 2021 Jun 17;10(6):543. doi: 10.3390/biology10060543 (PMC8233974; doi:10.3390/biology10060543)

Supplementary Table S1: Primers used in this study

Gene Symbol	Ref. Seq. ID	Forward Primer	Reverse Primer
mm Ech1	NM_016772	TCGCTACTGCACTCAGGATG	AGCAGCCAAGCCCATATCTA
mm Cpt2	NM_009949	TGCCCAGGCTGCCTATCCCTAAACT	GCTCCTTCCCAATGCCGTTCTCAAAAT
mm PPARα	NM_011144	CCTCAGGGTACCACTACGGAGTT	TCGCCGAAAGAAGCCCTTA
mm PGC1α	NM_008904	AGCCTCTTTGCCCAGATCTTC	CCATCTGTCAGTGCATCAAATGA
mm Cs	NM_026444	CGGGAGGGCAGCAGTATCGG	ACCACCCTCATGGTCACTATGGATG
mm Slc25a20	NM_020520	CCAACGCTGCCTGTTTCCTTGG	GCCAAGCCTGGAGCTCTGATCAC
mm Cox2	NC_005089	GCCGACTAAATCAAGCAACA	CAATGGGCATAAAGCTATGG
mm Cytb	NC_005089	CATTTATTATCGCGGCCCTA	TGTTGGGTTGTTTGATCCTG
mm Gcg	NM_008100	CAGGGCCATCTCAGAACC	GCTATTGGAAAGCCTCTTGC
mm Hbb	NM_001278161	GAAGCGATTCTAGGGAGCAG	GGAGCAGCGATTCTGAGTAGA
mm Pdk4	NM_013743	CCCTTTGGCTGGTTTTGGT	TTGGCGTAGAGACGAGAAATTG
mm Cd36	NM_ 001159555	GCAAAGAACAGCAGCAAAATC	TCCTCGGGGTCCTGAGTTAT
mm Acads	NM_007383	GCTGAGTGGTGCAGGCTTG	CCATTGGTGAAAGGGGTGATC
mm Acadm	NM_007382	TTGAGTTGACGGAACAGCAG	AGTTTGCACCCCTGTACACC
mm Acadl	NM_007381	TCACCACACAGAATGGGAGA	ACGCTTGCTCTTCCCAAGTA
mm Acadvl	NM_017366	TATCTCTGCCCAGCGACTTT	TGGGTATGGGAACACCTGAT

Supplementary Figure S1: Fat and Lean mass gain in control and HuR$^{m-/-}$ mice. A) Fat mass is plotted from 4-20 weeks of age for control (red) and HuR$^{m-/-}$ (blue) male mice fed LFD (squares and circles) or HFD (triangles and diamonds). **B)** Lean mass is plotted from 4-20 weeks of age for control (red) and HuR$^{m-/-}$ (blue) male mice fed LFD (squares and circles) or HFD (triangles and diamonds). **C)** Body Mass is plotted from 4-20 weeks of age for control (red) and HuR$^{m-/-}$ (blue) male mice fed LFD (squares and circles) or HFD (triangles and diamonds). N=14-19 for male animals. **D)** Fat mass is plotted from 4-20 weeks of age for control (red) and HuR$^{m-/-}$ (blue) female mice fed LFD (squares and circles) or HFD (triangles and diamonds). **E)** Lean mass is plotted from 4-20 weeks of age for control (red) and HuR$^{m-/-}$ (blue) female mice fed LFD (squares and circles) or HFD (triangles and diamonds). **F)** Body Mass is plotted from 4-20 weeks of age for control (red) and HuR$^{m-/-}$ (blue) female mice fed LFD (squares and circles) or HFD (triangles and diamonds). N=16-19 for female animals. Bottom asterisks and hashtags represent significance between genotypes fed LFD. Top asterisks and hashtags represent significance between genotypes fed HFD. * - P≤0.05; # - P≤0.1

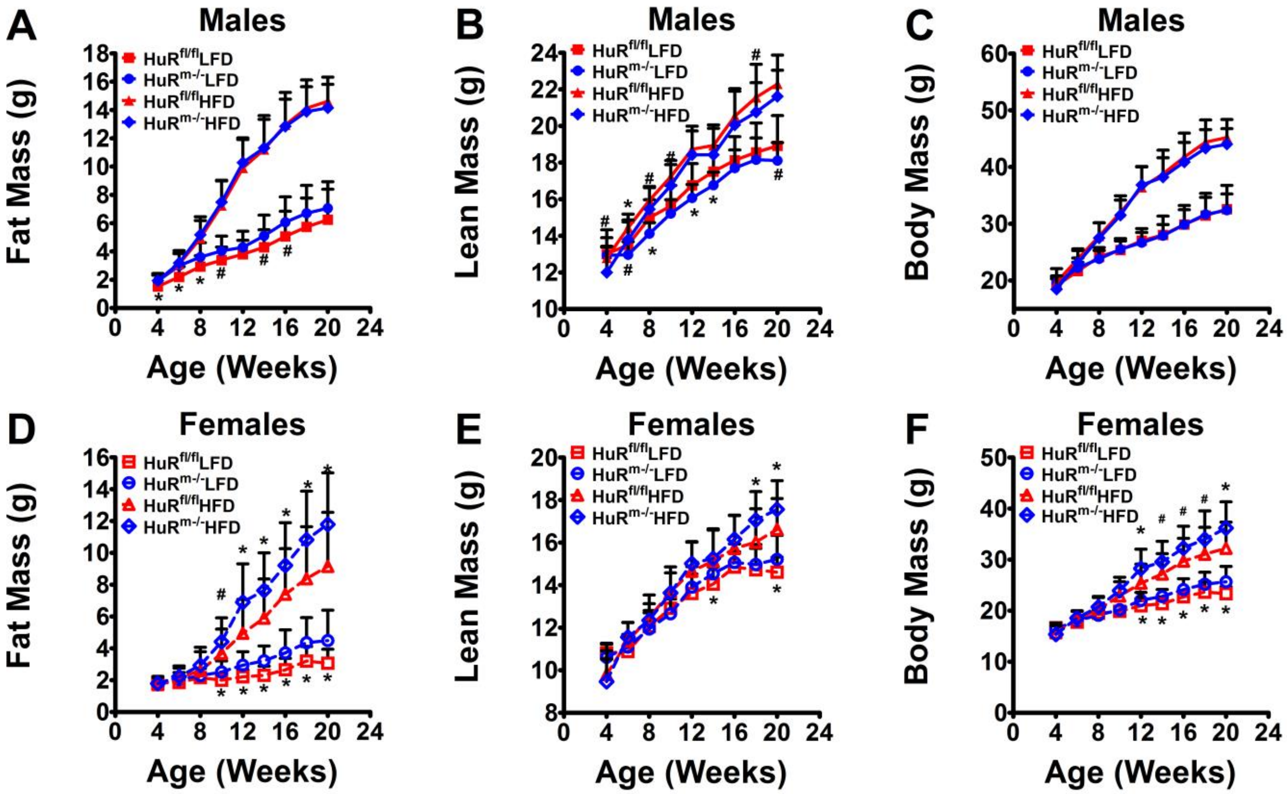

Supplementary Figure S2: Increased dietary fat intake increases fat mass in control and HuR$^{m-/-}$ mice. A) Fat mass at 20 Weeks of age as a function of dietary fat intake for control (red) and HuR$^{m-/-}$ (blue) male mice. **H)** Fat mass at 20 Weeks of age as a function of dietary fat intake for control (red) and HuR$^{m-/-}$ (blue) female mice.

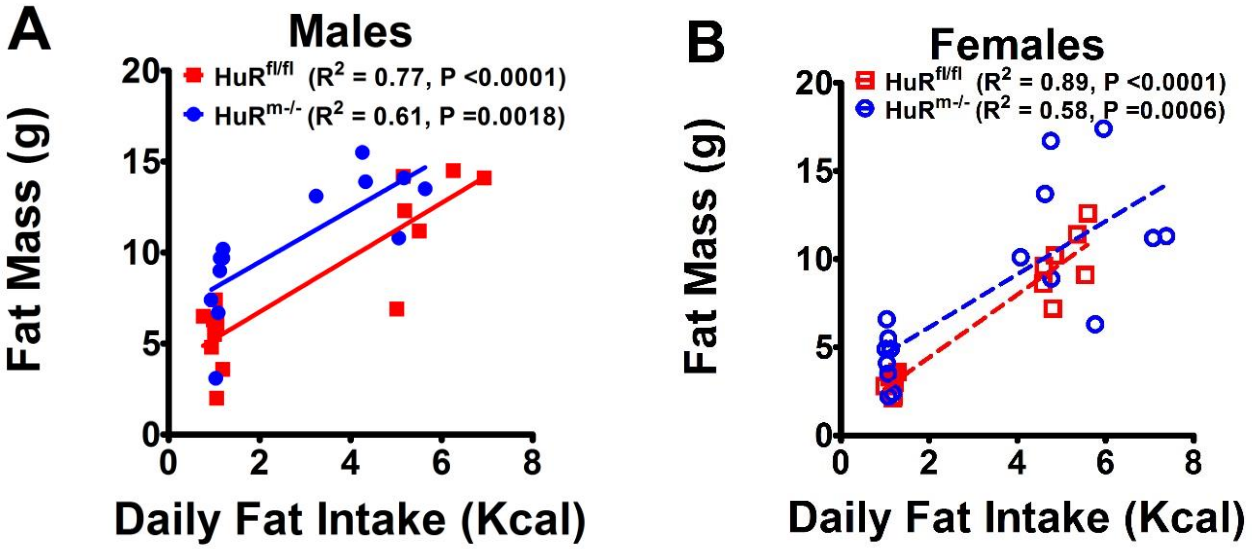

Supplementary Figure S3: Energy Expenditure graphs for mice in this study. A) Energy Expenditure at 20-22 weeks of age for male control (red) or HuR$^{m-/-}$ (blue) mice fed LFD (N=8). **B)** Energy Expenditure at 20-22 weeks of age for male control (red) or HuR$^{m-/-}$ (blue) mice fed HFD (N=6-8). **C)** Energy Expenditure at 20-22 weeks of age for female control (red) or HuR$^{m-/-}$ (blue) mice fed LFD (N=7-8). **D)** Energy Expenditure at 20 weeks of age for female control (red) or HuR$^{m-/-}$ (blue) mice fed HFD (N=8).

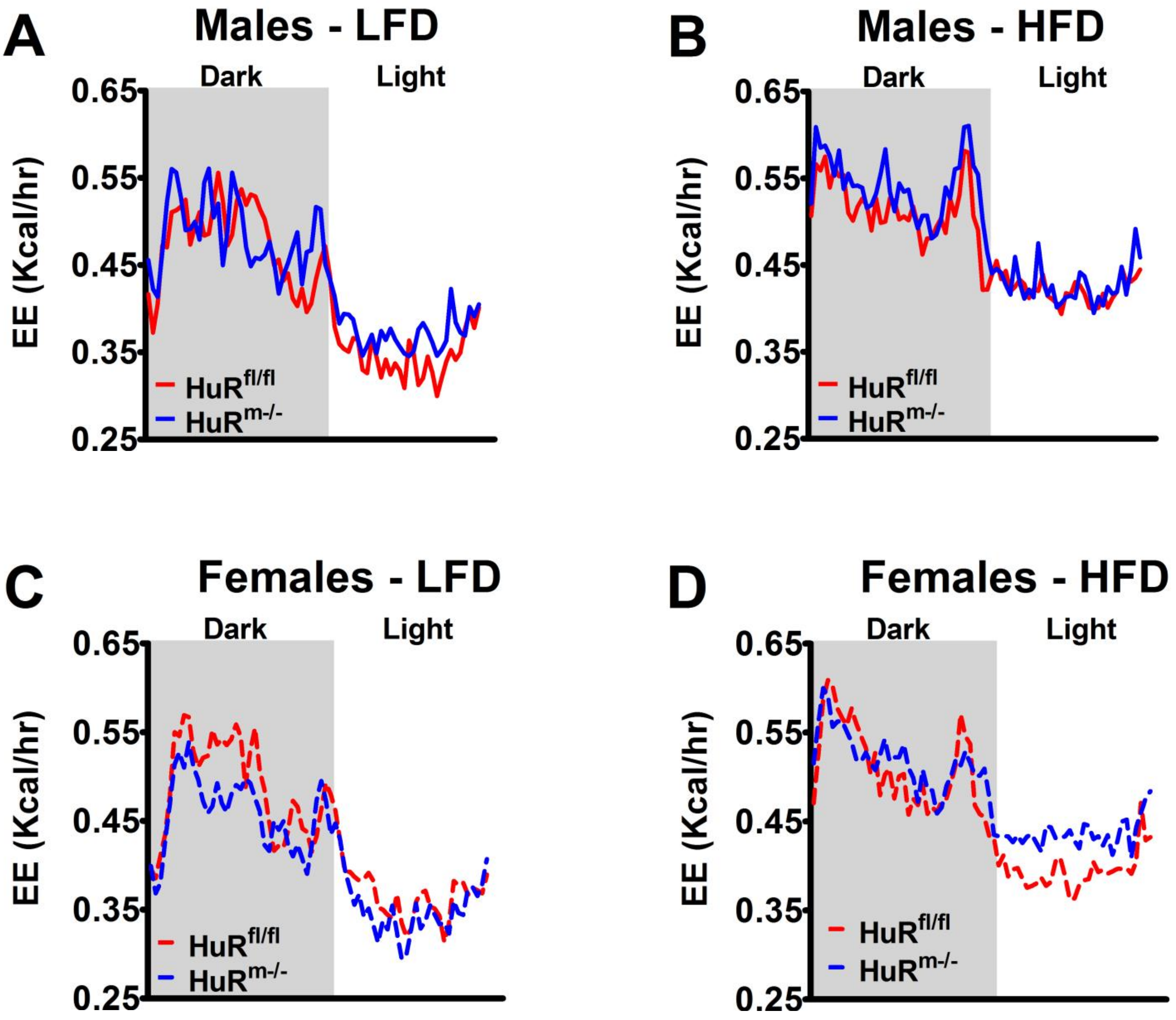

Supplementary Figure S4: Area under the curve for GTT and ITT in this study. A) GTT at 20 weeks of age for male control (red) or HuR$^{m-/-}$ (blue) mice fed HFD (N=18). **B)** ITT at 20 weeks of age for male control (red) or HuR$^{m-/-}$ (blue) mice fed HFD (N=18). **C)** GTT at 20 weeks of age for male control (red) or HuR$^{m-/-}$ (blue) mice fed LFD (N=14-16). **D)** ITT at 20 weeks of age for male control (red) or HuR$^{m-/-}$ (blue) mice fed LFD (N=10-16). **E)** GTT at 20 weeks of age for female control (red) or HuR$^{m-/-}$ (blue) mice fed HFD (N=19). **F)** ITT at 20 weeks of age for female control (red) or HuR$^{m-/-}$ (blue) mice fed HFD (N=19). **G)** GTT at 20 weeks of age for male control (red) or HuR$^{m-/-}$ (blue) mice fed LFD (N=12-16). **H)** ITT at 20 weeks of age for male control (red) or HuR$^{m-/-}$ (blue) mice fed LFD (N=14-16).

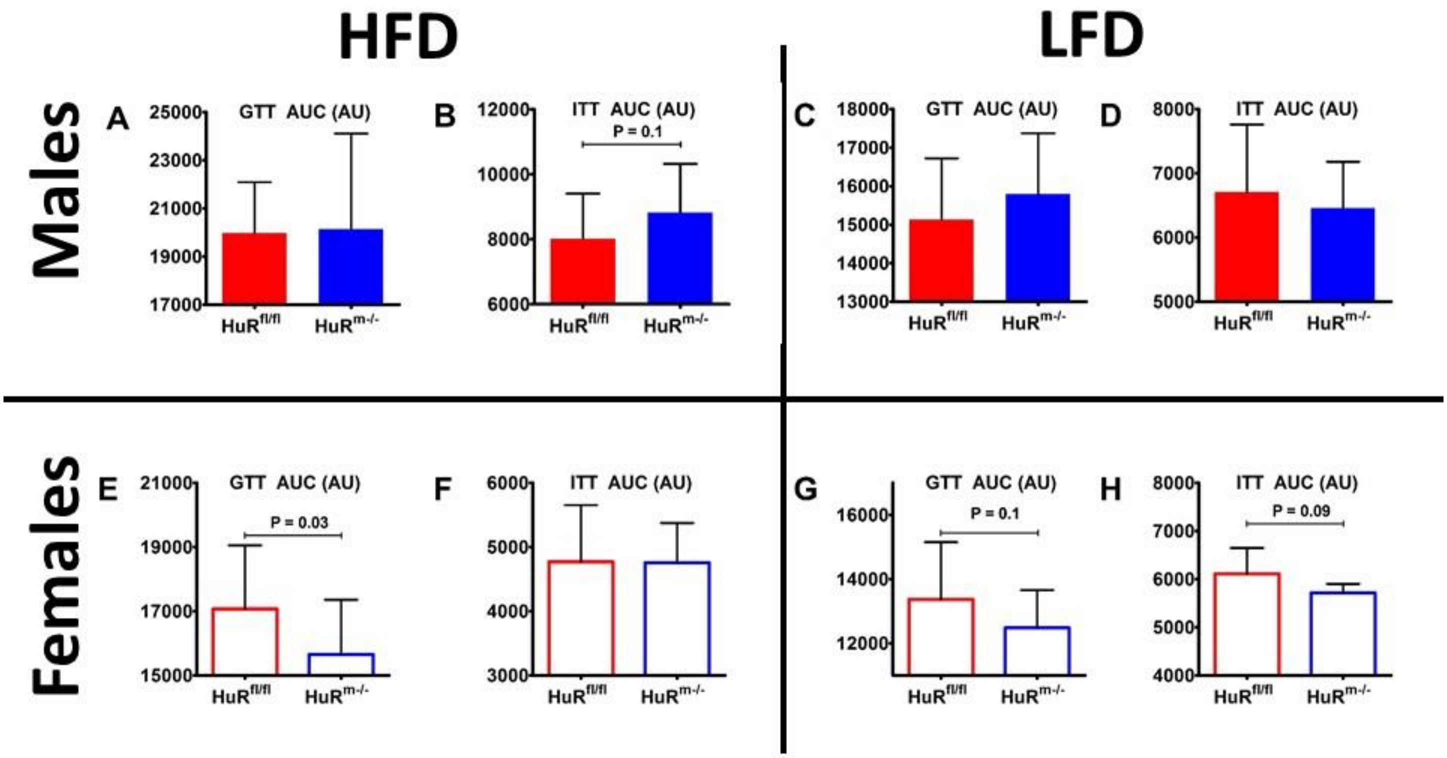

Supplementary Figure S5: HOMA-IR calculated from 4-hour fasted glucose and insulin from animals in this study. A) HOMA-IR shown at 20 weeks of age for male control (red) or HuR$^{m-/-}$ (blue) mice (N=7-8). **B)** HOMA-IR shown at 20 weeks of age for female control (red) or HuR$^{m-/-}$ (blue) mice (N=7-8).

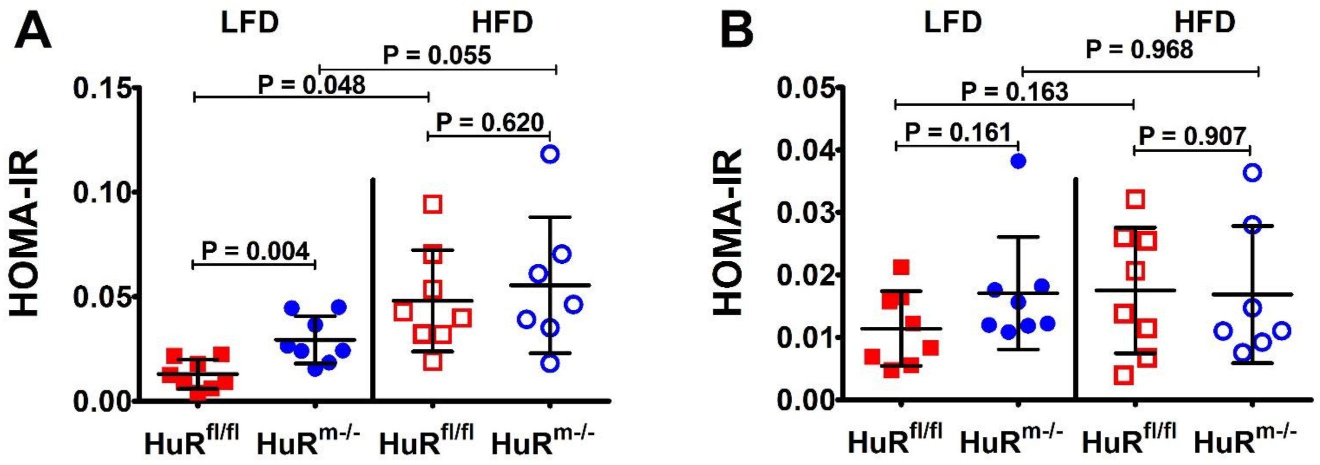

Supplementary Figure S6: Fat pad weights and body temperature for HuR$^{m-/-}$ and control mice A-B) combined fat pad weights are shown for control and HuR$^{m-/-}$ males (A) and females (B). Fat pad weights are the summation of inguinal (i-pink), gonadal (g-teal), and retroperitoneal (rp-lilac) depots (N=6-10). **C-D)** Brown adipose tissue weights are shown for control and HuR$^{m-/-}$ males (C) and females (D) (N=6-10). **E)** Body temperatures are shown for control (red) and HuR$^{m-/-}$ (blue) males (closed) and females (open) fed low fat diet.

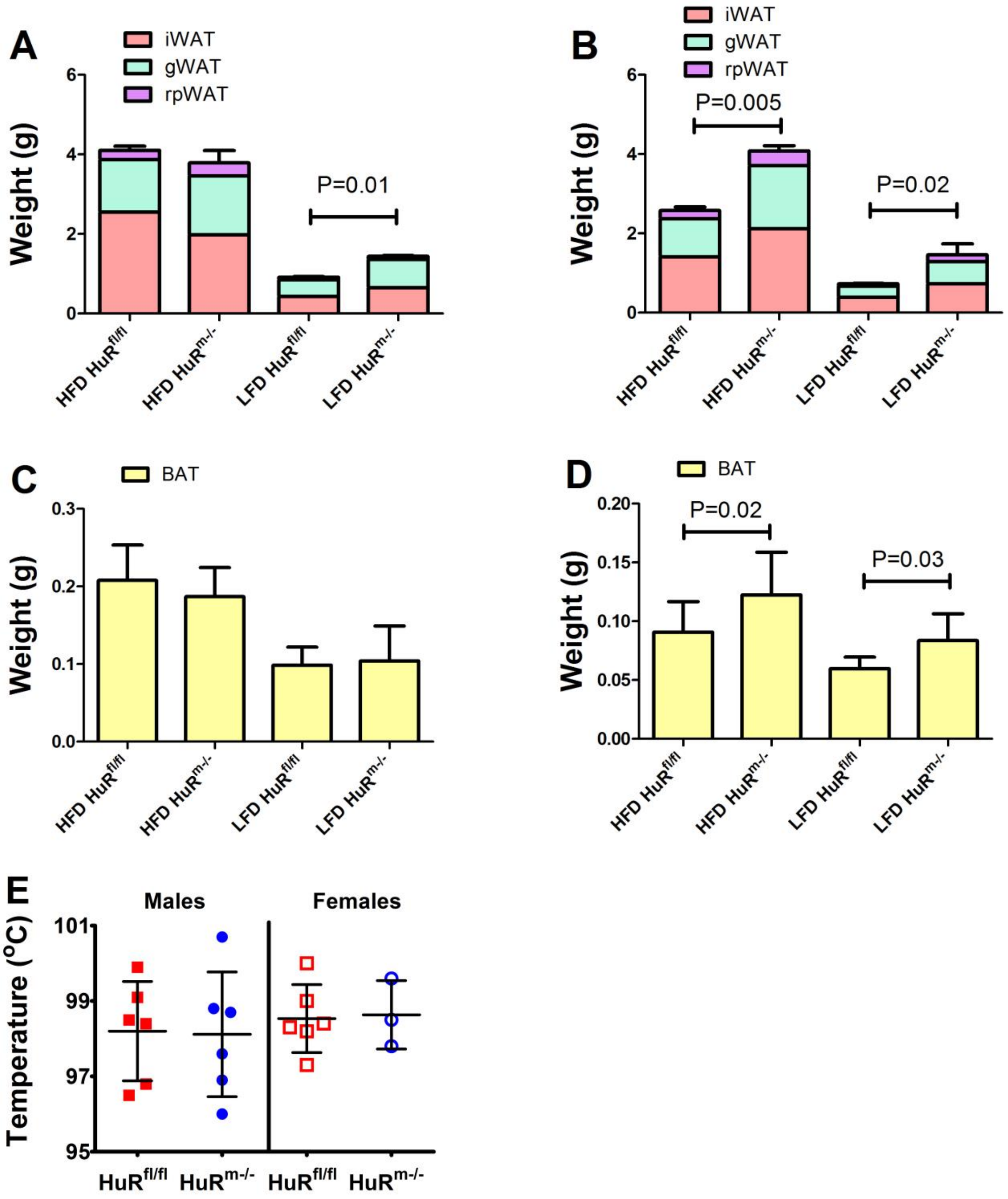

Supplement: Supplementary file 1 [file biology-10-00543-s001.zip › biology-1256195-supplementary.pdf]
